# The T-Cell Response to Lipid Antigens of *Mycobacterium tuberculosis*

**DOI:** 10.3389/fimmu.2014.00219

**Published:** 2014-05-22

**Authors:** Gennaro De Libero, Lucia Mori

**Affiliations:** ^1^Singapore Immunology Network, Agency for Science, Technology and Research (A*STAR), Singapore, Singapore; ^2^Experimental Immunology, Department of Biomedicine, University Hospital Basel, Basel, Switzerland

**Keywords:** CD1, lipid antigens, tuberculosis, T-cells, antigen presentation

## Abstract

T-cells recognize lipid antigens presented by dedicated antigen-presenting molecules that belong to the CD1 family. This review discusses the structural properties of CD1 molecules, the nature of mycobacterial lipid antigens, and the phenotypic and functional properties of T-cells recognizing mycobacterial lipids. In humans, the five CD1 genes encode structurally similar glycoproteins that recycle in and thus survey different cellular endosomal compartments. The structure of the CD1-lipid-binding pockets, their mode of intracellular recycling and the type of CD1-expressing antigen-presenting cells all contribute to diversify lipid immunogenicity and presentation to T-cells. Mycobacteria produce a large variety of lipids, which form stable complexes with CD1 molecules and stimulate specific T-cells. The structures of antigenic lipids may be greatly different from each other and each lipid may induce unique T-cells capable of discriminating small lipid structural changes. The important functions of some lipid antigens within mycobacterial cells prevent the generation of negative mutants capable of escaping this type of immune response. T-cells specific for lipid antigens are stimulated in tuberculosis and exert protective functions. The mechanisms of antigen recognition, the type of effector functions and the mode of lipid-specific T-cell priming are discussed, emphasizing recent evidence of the roles of lipid-specific T-cells in tuberculosis.

## Introduction

The discovery that T lymphocytes recognize lipid molecules as antigens (1) was a breakthrough in cellular immunology that opened unexpected horizons in the field of immune response to microbes, to cancer cells, and in autoimmunity.

Despite main structural differences with peptide antigens, lipid antigens are presented to T-cells following general rules that closely resemble those implicated in the presentation of peptide antigens. Indeed, only lipids forming stable complexes with dedicated antigen-presenting molecules, which belong to the CD1 protein family (2), are capable of interacting with the T-cell receptor (TCR) and may induce the activation of specific T-cells.

Many different types of lipids behave as antigens capable of stimulating specific T-cells and *Mycobacterium tuberculosis* is the bacterium with the largest number of antigenic lipids identified so far. Mycobacteria are characterized by a complex cellular envelope composed of a variety of unique lipids (3), which have important functions in survival of mycobacterial bacilli (4). Envelope lipids are also implicated in tissue-specific replication (5) and controlled penetration of engulfing cells (6) and in escaping immune response with different mechanisms (7). Therefore, Mycobacteria are reluctant in changing the lipid composition of their envelope. Mycobacterial lipids, glycolipids, and lipopeptides may bind to and form stable complexes with different CD1 molecules, and may interact with specific TCR.

The generation of stable CD1–lipid antigen complexes mostly occurs within the infected cell and follows a series of well coordinated events. These include the transport of lipid antigens by lipid chaperones in cellular membranes and endosomal compartments, the extraction of lipids from membranes, their processing, and subsequent insertion in the hydrophobic pockets of CD1 molecules. Stable CD1–lipid complexes are then routed to the plasma membrane where they become available for T-cell recognition (8).

Mycobacteria have evolved a series of tactics to interfere with lipid antigen presentation, showing the surprising capacity of inhibiting and hijacking different protective immune mechanisms. The existence of these evasion strategies is the indirect evidence that lipid-specific immune response represents an important mechanism of defense in tuberculosis. How lipid-specific T-cells participate in controlling mycobacterial infections is an important field of investigation in many laboratories that is providing new information on protective immune mechanisms and is suggesting novel approaches to vaccine development.

This chapter describes the nature, structure, and function of mycobacterial lipid antigens, the mechanisms of their recognition by T-cells, the biological properties of CD1 antigen-presenting molecules and the rules that govern presentation of lipids to T-cells. The relevance of lipid-specific T-cells in tuberculosis is also discussed.

## CD1 Antigen-Presenting Molecules

In humans five genes encode different CD1 isoforms, which are classified into three families (group 1, 2, and 3). CD1a, b, and c molecules belong to the group 1 family, whereas CD1d and CD1e are the only members of group 2 and 3, respectively. The separation into three families follows important structural differences, cellular and tissue distribution of each CD1 protein. CD1a, b, and c are expressed on CD4, CD8 double positive thymocytes, and on peripheral antigen-presenting cells (APC), including dendritic cells (DC), monocytes, and a subset of B cells. Each of these molecules can be uniquely expressed. For example, CD1a, but not CD1b and c are expressed by Langerhans cells in the skin and represent a marker distinguishing this cell population. CD1b is expressed by a population of monocytes and is upregulated during differentiation into DC. CD1c is expressed on a large fraction of human B cells. CD1d is instead more extensively expressed on both hematopoietic and non-hematopoietic cells.

CD1e is the only CD1 protein that is not expressed on the plasma membrane and remains confined within the Golgi complex and late endosomes/lysosomes. All CD1 molecules are expressed by professional APC such as DC.

The structures of all CD1 molecules have been solved and showed important differences that illustrate their unique antigen-binding capacities.

## Structure of CD1 Molecules and Repertoire of Presented Lipid Antigens

CD1 molecules have structures similar to those of MHC class I molecules. They are glycoproteins composed of a heavy chain non-covalently associated with β2-microglobulin. The heavy chain is made of three domains. The α1 and α2 domains, which assemble together in the membrane distal part of the molecule, are characterized by two anti-parallel α helices positioned above a β sheet plate. The α3 domain instead is membrane-proximal and associates with β2-microglobulin. It also continues with the transmembrane domain and a short intracytoplasmic tail. Each CD1 molecule shows a portal located in between the α1 and α2 helices, which allows access to two to four pockets. The pockets are surrounded by hydrophobic amino acids, thus representing ideal places to allocate the apolar alkyl chains of lipid antigens.

## CD1a

Each CD1 molecule shows unique structural features.

CD1a has one portal located between the two α helices, which is connected with two pockets, called A′ and F′ (a nomenclature similar to that of MHC molecules). Around the portal a few charged amino acids stabilize and properly orient the antigen headgroup for TCR recognition. The F′ pocket has an incomplete roof thus being partially open on the surface of CD1a. The mode of lipid-binding of CD1a is unique, since the open F′ pocket permits ready insertion and replacement of lipid antigens. This feature optimally complements another unique characteristics of CD1a, i.e., its recycling through early endosomal stations (9, 10). Early endosomal compartments are slightly acidic environments that do not induce main conformational changes to the structure of recycling proteins. In addition, lipid transfer proteins that facilitate lipid antigen loading on CD1 molecules are localized in other endosomal compartments. Therefore, an open F′ pocket seems a natural evolution of CD1a to facilitate otherwise unfavorable conditions for loading of lipid antigens.

The co-crystal of CD1a with lipid antigens have also provided important information on another consequence of the unique open F′ pocket structure.

The CD1a-sulfatide structure revealed that the sphingosine occupies the A′ pocket whereas the fatty acid chain protrudes in the interface of the A′ and F′ pockets. The headgroup of sulfatide, made by a galactose and a sulfate is anchored in the A′–F′ junction and partially occupies the upper part of the F′ pocket in a position ideally suited for TCR recognition (11).

The crystal structure of CD1a with lipopeptide dideoxymycobactin (DDM) instead showed that while the single alkyl chain was inserted deep within the A′ pocket, the two peptidic moieties of the antigen were lying partially in the F′ pocket and at the surface of CD1a, an optimal position for recognition by the TCR (12).

## Mycobacterial Lipid Antigens Presented by CD1a

The overall size of CD1a grove is limited to 1350 Å^3^, thus precluding binding of lipid antigens of large size. So far, only DDM has been identified as mycobacterial antigen presented by CD1a (13).

Dideoxymycobactin is the precursor of mycobactin, a siderophore with iron scavenging properties. The structure of DDM shows a complex head group made of unusual amino acids synthesized by non-ribosomal mycobacterial enzymes. The lysine of headgroup is linked to a single alkyl chain, which is 20 carbons long. One clone specific for CD1a–DDM was isolated and when it was tested the length and saturation of the DDM alkyl chain appeared very important for T-cell stimulation. This behavior was fully explained by the structural resolution of the CD1a–DDM complex, which showed a deep insertion of the acyl chain in the CD1a A′ pocket. As this pocket is closed at one end, the insertion of too short or too long acyl chains determines a different positioning of the peptidic residues, thus directly influencing the interaction with the TCR. This mode of binding led the authors to discuss a role as a ruler of the A′ CD1a pocket (12). During their intracellular growth, mycobacteria increase mycobactin synthesis to increase their capacity of iron capture. Thus presentation of DDM may be an efficient mechanism to identify cells harboring living mycobacteria.

## CD1b

The molecule CD1b is expressed by activated monocytes and DC. CD1b presents most of the identified mycobacterial antigens and follows unique recycling and lipid loading rules. Within the group 1 CD1 family, CD1b has the largest pocket (~2200 Å^3^) and is made by four interconnected pockets, which confer unique lipid antigen-binding properties. A large portal is located between the two α helices that is connected with the A′, C′, and F′ pockets. The A′ pocket continues into the F′ pocket through a tunnel called T′ pocket. The combination of the four pockets with the large size, allows CD1b to bind lipid antigens with very long alkyl chains, such as the mycobacterial mycolyl chains that can reach a length of 80 carbons. The structure of CD1b has been solved thus revealing how CD1b can accommodate either very long or short lipid antigens (14–17). Mycolic acid (MA), an antigen with long alkyl chain that can contain up to 80 carbons occupies the A′, T′, and F′ pockets, whereas the mycobacterial diacylated sulfoglycolipids (SGL) occupy the A′ and C′ pockets with their two alkyl chains. This flexibility in lipid-binding raised the question of why the CD1b T′ pocket does not collapse when antigens with short alkyl chains are bound. The reason for this stability was found when a soluble CD1b molecule produced in eukaryotic cells and physiologically refolded *in vivo* was crystallized. This molecule showed the presence of different spacers within the T′ pocket, thus representing a filling lipid preventing the collapse of the molecule (18).

## Mycobacterial Lipid Antigens Presented by CD1b

Mycolic acids were the first antigens, which were identified as lipids capable of stimulating specific T-cells (19). These long and highly hydrophobic lipids represent very important components of the cell wall of several bacteria, including Mycobacteria, Corynebacteria, and *Nocardia*. MA are unique fatty acids of high-molecular-weight, with α-alkyl, β-hydroxyl groups. They may be present as such or as bound esters of sugars like arabinogalactan and trehalose. Activation assays using different types of MA, showed that the hydroxyl and carboxyl groups may represent important structural features sensed by T-cells (19).

Mycolic acid may also be considered as scaffold molecules that form stimulatory antigen when attached to other molecules. For example, glucose monomycolate (GMM) is capable of stimulating specific T-cells (20). In GMM, a glucose residue is attached to MA and is critical for T-cell recognition as the stereoisomers mannose- or galactose-monomycolate are not or poorly stimulatory. T-cells also discriminate the type of glycosidic bond, as GMM with glucose bound with 6-hydroxyl to MA is stimulatory, whereas glucose-3-monomycolate is not stimulatory (21).

Another antigen with modified MA is glycerol monomycolate (GroMM) (22) that is made of a glycerol moiety attached to a MA and is also produced by Mycobacteria, Corynebacteria, and *Nocardia*. The glycerol hydroxyl moiety and the fatty acid length of GroMM influence T-cell response. MA-related lipids are presented only by CD1b as this is the only CD1 molecule with an open T′ pocket allowing the insertion of long fatty acids.

A second family of lipid antigens presented by CD1b is those sharing a phosphatidyl-myo-inositol (PI). Phosphatidylinositol mannoside (PIM) and lipoarabinomannan (LAM) are two lipid antigens presented by CD1b (23). LAM and PIM share a core region, where the mannoses linked to PI are the stimulatory antigens. LAM contains a terminal part with many arabinoses in some instances capped with mannoses. Lipomannan (LM), which is similar to LAM, but does not contain arabinose, can also stimulate specific T-cells (23). T-cells discriminate lipoglycans made in different bacteria. T-cells recognizing LAM from *Mycobacterium leprae*, do not recognize LAM from *M. tuberculosis* (23). These LAM differ in the number and branching points of mannoses present in the core that are probably involved in interacting with the TCR.

All lipoglycans are too big to allocate within the CD1b pockets and interact with T-cells. They require internalization and processing in late endosomes (24). PIM_6_, which is the hexamannosylated form of PIM, also requires processing with shortening of the hexamannoses in order to stimulate specific T-cells (25). Another aspect that is not clear yet is whether these antigens also require processing by lipases. Indeed naturally occurring PIM is characterized by four alkyl chains and they need *ad hoc* cleavage before forming stable complexes with CD1b (our unpublished data).

A third family of CD1b-presented lipid antigens is that of SGL (26). SGL are localized in the outer part of cell envelope and their abundance was associated with strain virulence (27), although mutant strains revealed that sulfolipid deficiency does not significantly affect the replication, persistence, and pathogenicity of *M. tuberculosis* H37Rv in mice and guinea pigs or in cultured macrophages (28). SGL contain a trehalose 2′ sulfate core, acylated by two to four fatty acids. This antigen isolated from virulent mycobacteria strains proved to a be a mixture of structurally related diacylated SGL (Ac_2_SGL) containing hydroxyphtioceranoic and palmitic or stearic acid, both linked to the glucose without the sulfate in the trehalose. The tri- and tetra-acylated forms were not immunogenic, thus revealing that APC do not have the proper lipase apparatus to digest these forms and generate the diacylated immunogenic forms. Removal of the sulfate group completely abolished T-cell activation (26), suggesting that the fine antigen specificity of the TCR is also influenced by the strong negative charges of sulfate.

The structure of CD1b–SGL complex has been solved and showed a unique mode of interaction of this lipid antigen with the CD1b (17). Comparison of the CD1b crystals with and without SGL showed that upon antigen-binding, the endogenous spacers of CD1b, which consist of a mixture of diradylglycerols, move considerably within the lipid-binding groove. Spacer displacement was accompanied by F′ pocket closure and an extensive rearrangement of residues exposed to T-cell receptors. Such structural reorganization resulted in reduction of the A′ pocket capacity and led to incomplete embedding of the methyl-ramified portion of the phthioceranoyl chain of the antigen, explaining why such hydrophobic motifs are critical for T-cell receptor recognition. Mutagenesis experiments supported the functional importance of the observed structural alterations for T-cell stimulation. Thus, CD1b is a very plastic molecule, capable of important structural rearrangements that combine spacer repositioning and ligand-induced conformational changes. Both these changes endow CD1b with the capacity to present a broad range of structurally diverse antigens and to focus the TCR only on the antigen-filled CD1b molecules.

## CD1c

The CD1c molecule is characterized by unique features that contribute to the important biological features of this CD1 isotype.

The central portal located in between the two alpha helices allows protrusion of the hydrophilic moiety of bound antigen, which is presumably involved also in contacting the TCR. This portal is connected with the A′ and F′ pockets. The A′ pocket may accommodate the alkyl chain of bound antigen (29). The A′ pocket is open to the external part of CD1c with an exit portal located underneath the α1 helix. Interestingly, the F′ pocket of CD1c showed an open cavity, which is similar to the antigen-binding groove of MHC-peptide-binding molecules and in some respects to the F′ pocket of CD1a. The F′ pocket is also open to the exterior with a second portal called E′. Whether these additional portals facilitate binding of very long alkyl chains, remains unclear, although this type of antigen interaction is energetically unfavorable.

CD1c is also characterized by a unique type of intracellular recycling. It may recycle in deep endosomal compartments, where low pH may facilitate partial CD1 unfolding and antigen loading. Furthermore, in these compartments dedicated lipid antigen-binding proteins (LBP) are present, which behave as lipid chaperones and are involved in lipid antigen-binding, transport, and loading on CD1 molecules. This mode of antigen loading resembles that of lipids on CD1b and CD1d. In addition, some CD1c molecules recycle through the early endosomal compartments, where the most acidic pH is 6.5 and probably no LBP are present. In this cellular compartment, the partially open structure of the F′ pocket might facilitate antigen exchange and binding.

## Mycobacterial Lipid Antigens Presented by CD1c

Both the unique portals and structure of the F′ pocket explain the promiscuous antigen-binding behavior of CD1c. This molecule may present lipid antigens containing one alkyl chain (30), two alkyl chains (31), or lipopeptides formed by one alkyl chain linked to a short peptide (32). Furthermore, CD1c stimulates a population of T-cells expressing the gamma and delta chains of the TCR (TCR γδ) (33, 34). As the described CD1c-restricted TCR γδ cells are autoreactive, it is very probable that CD1c presents a self-antigen, which remains unknown.

Recently, we have identified the novel self lipid methyl-lysophosphatidic acids (mLPA), not described before, which accumulates in myeloid and lymphoid leukemia cells and induces a CD1c-restricted response directed against these human leukemias (35). mLPA-specific T-cells efficiently kill CD1c^+^ acute leukemia cells, and protect immunodeficient mice against CD1c^+^ human leukemia cells and poorly recognize non-transformed CD1c-expressing cells. Whether similar types of lipids are produced by mycobacteria or accumulate in infected cells during mycobacterial infection and induce CD1-restricted T-cell responses is matter of current investigation.

Mannosyl β-1-phosphomycoketides (MPM) were the first CD1c-restricted lipid antigens (30). MPM contain a single fully saturated alkyl chain with methyl branches at every fourth carbon. MPM are synthesized by Mycobacteria infecting human cells, including *M. tuberculosis*, BCG, and *Mycobacterium avium*, and not by rapidly growing saprophytes, including *Mycobacterium phlei, Mycobacterium fallax*, and *Mycobacterium smegmatis* (36). The structure of MPM is similar to that of phosphodolichols that in mammalian cells function as carbohydrate donors in glycan synthesis. However, differently from phosphodolichols they are not synthesized through an isoprenoid-independent pathway. Instead, the synthesis of MPM is accomplished by the enzyme Pks12 and requires the sequential condensation of malonate and methylmalonate units. Probably, these polyketides are involved in bacterial intracellular growth and in mannose transmembrane transport, according to their structural similarity with eukaryotic phosphodolichols. As found with other glycolipid-specific T-cells, also MPM-specific T-cell recognition is sensitive to structural changes in the hydrophilic and hydrophobic parts of MPM. Antigen recognition occurs when glucose and not mannose is present. Furthermore, the saturated α-prenyl like unit and the length of the prenyl chain are important for recognition. Moreover, variation in the number, length, and saturation of alkyl chains, and the precise chemistry and chirality of the lipid head group, also exert dominant influences on antigenicity (30, 36, 37), probably by affecting the mode of CD1 binding and interaction with the TCR (38).

## CD1d

Like other CD1 molecules, also CD1d contains antigen-binding pockets, which are much deeper than the groove of peptide-binding MHC molecules. The two pockets are constituted by a non-polar or hydrophobic surface. The pockets converge in a portal open on the surface of CD1d and located in between the two anti-parallel helices. A series of TCR–CD1d–lipid antigen co-crystals have shown the importance of the polar antigen residues, which provide minimal but essential interactions with the TCR (39–42).

The A′ pocket of CD1d is closed and allows the insertion of alkyl chains with a defined length. In the presence of lipid antigens with too short alkyl chains, small fatty acids may also be present, which contribute to filling the entire A′ pocket probably behaving as rulers preventing CD1d collapse (43).

CD1d is expressed on mostly all hematopoietic cells. It is also present on the surface of gut epithelial cells, adipocytes, and some keratinocytes. Most of the cells retain a large fraction of properly assembled CD1d in late endosomes and lysosomes, in a manner similar to that of MHC class II molecules. Upon synthesis, CD1d rapidly traffics to the plasma membrane and then initiates a fast intracellular recycling. A fraction of CD1d molecules is assembled together with the invariant chain that promotes a direct traffic to late endosomes (44). The mode of intracellular trafficking of CD1d is dependent on the unique intracytoplasmic tail that facilitates the interaction with the AP3 proteins (45). Indeed, this association is necessary to properly load lipid antigens intracellularly (46).

Many microorganisms modulate surface CD1d, including *M. tuberculosis*. Several studies have outlines different modulation capabilities according to the type of cell, the triggered surface receptor, and the possible involvement of soluble factors. While several viruses, including vaccinia, herpes simplex, hepatitis, and lymphocytic choriomeningitis viruses decrease CD1d expression in myeloid cells (47–49), infections with *Salmonella typhimurium* or *E. coli* increase the plasma membrane display of CD1d (50). *M. tuberculosis* infection or mycobacterial lipids also upregulate CD1d on bone-marrow-derived macrophages (51). Instead *M. tuberculosis* infection prevents the upregulation of CD1d in monocytes that differentiate to DC (52, 53).

## Mycobacterial Lipid Antigens Presented by CD1d

CD1d-restricted T-cells are classified in two major groups. The most widely investigated are invariant natural killer T (iNKT) cells, which express an invariant human Vα24Jα18 or mouse Vα14Jα18 TCR chain. These cells recognize with high affinity the α-galactosylceramide (αGalCer) lipid extracted from the marine sponge *Agelas mauritianus* (54). iNKT cells are highly conserved in many species, thus suggesting that they exert important immunological functions conserved during evolution. iNKT cells also recognize other non-microbial antigens, including phosphatidylinositol (PI), phosphatidylglycerol (PG), and phosphatidylethanolamine (PE), although with a weak affinity compared to the strong agonist αGalCer (55). Lysophosphatidylcholine, a lipid generated by lipid-dependent signaling pathways, may also stimulate iNKT cells (56). Other CD1d-presented antigens are the ether-bonded mono-alkyl glycerophosphates that together with the precursors and degradation products of plasmalogens are required for the proper selection and maturation of iNKT cells in the thymus (57).

Very initial studies suggested that a tetramannosylated PIM form (PIM_4_) present in a fraction of lipids purified from *Mycobacterium bovis* BCG was a potential CD1d-presented iNKT cell antigen (58). These findings have not been confirmed in subsequent studies and the potential role of PIM_4_ as CD1d-presented antigen to iNKT cells still awaits confirmation. While CD1d mycobacterial antigens have not been identified so far, other bacteria produce strong iNKT stimulatory lipids, including the glycolipids with diacylglycerol moieties of *Borrelia burgdorferi* and *Streptococcus pneumoniae* (59, 60), and the glycosphingolipids present in the cell walls of bacteria belonging to the genera *Sphingomonas* and *Novosphingobium* (61). Surface glycophospholipids of the protozoal parasites *Leishmania* species are also iNKT stimulatory antigens (62). iNKT-deficient mice are more susceptible to *Leishmania* infection, indicating that iNKT cells have important functions in protection. However, it is still unknown how *Leishmania* lipophosphoglycan (LPG) stimulate these T-cells. While LPG binds to CD1d, CD1d–LPG complexes do not stimulate iNKT cells. DC pulsed with LPG can induce IFNγ release by iNKT cells, suggesting that LPG stimulates the production of endogenous lipid ligands, which in turn activate iNKT cells. This is not a remote possibility, since bacterial infections may induce important changes in the lipid metabolism of infected cells. This, in turn, may induce accumulation of endogenous lipids that stimulate T-cells restricted by either group 1 or group 2 CD1 molecules (63–65).

A second group of CD1d-restricted T-cells utilized non-invariant TCR and resemble other T-cells involved in classical adaptive immune responses. These T-cells recognize PG, diphosphatidylglycerol (DPG or cardiolipin), and PI from *M. tuberculosis* or *Corynebacterium glutamicum* as microbial antigens (66). Importantly, the same lipid molecules are present also in mammalian cells and thus they represent self-antigens. Thereby, the increased availability of these self-antigens within APC might also contribute to the activation of CD1d-restricted T-cells cross-reactive with microbial antigens.

## CD1e

CD1e is a unique type of CD1 molecule as it remains intracellular and therefore cannot be considered a *bona fide* antigen-presenting molecule (67). CD1e is mostly expressed by DC where it accumulates in the Golgi stack of immature cells as pre-protein (68). Upon maturation, CD1e is ubiquitinated (69) and traffics to the lysosomes where it is cleaved in two places. In the amino-terminal region a propeptide is cleaved, that is responsible for the assembly of CD1e with β2-microglobulin. Upon its removal CD1e becomes fully active (70). CD1e is also cleaved in its membrane-proximal region, thus becoming a mature soluble protein. CD1e is apparently not secreted and does not accumulate in other intracellular compartments. These features make CD1e similar to the HLA-DM and HLA-DO molecules, which are also not expressed on the plasma membrane of APC. The acid pH of lysosomes promotes a partial alteration of CD1e structure together with the binding of lipid antigens through increased hydrophobic and ionic interactions (71).

The crystal structure of CD1e revealed a groove less intricate than in other CD1 proteins, with a significantly wider portal. The water-exposed CD1e groove probably allows loose contacts with lipid antigens as supported by the finding that lipid association and dissociation processes were faster with CD1e than with CD1b (72).

All these structural features support the function of CD1e as a LBP with the exquisite capability of facilitating lipid antigen presentation by other CD1 molecules. Indeed, CD1e mediates *in vitro* the transfer of lipids to CD1b and the displacement of lipids from stable CD1b-antigen complexes (72). CD1e is also required for the proper processing of complex mycobacterial lipid antigens such as the PIM_6_ molecules. In the presence of CD1e, PIM_6_ is cleaved to PIM_2_ and is loaded on CD1b (25). CD1e may also positively or negatively affect lipid presentation by CD1b, CD1c, and CD1d (73). This effect is achieved by a rapid formation of CD1–lipid complexes in the presence of CD1e, and also by an accelerated turnover of formed CD1–lipid antigen complexes. These effects maximize and temporally narrow CD1-restricted responses. CD1e is therefore an important modulator of both group 1 and group 2 CD1-restricted responses influencing the lipid antigen availability as well as the generation and persistence of CD1–lipid complexes.

## T-Cells Recognizing Mycobacterial Lipid Antigens

T-cells stimulated by lipid antigens are divided into two categories according to their functional behavior and mechanism of antigen recognition. T-cells restricted by group 1 CD1 molecules and type II NKT cells are considered as arm of adaptive T-cell responses, while CD1d-restricted type II NKT or iNKT cells are considered as the typical representative of innate T-cell responses.

Lipid-specific T-cells that are restricted by group 1 CD1 molecules are present as naïve T-cells and expand after encountering the antigen, mostly during mycobacterial infections or BCG vaccination (26). They expand and may also differentiate into classical memory T-cells (74). These studies have been performed using human samples, as small rodents do not express group 1 CD1 molecules. Upon antigen recognition, lipid-specific T-cells release pro-inflammatory cytokines and may also kill intracellular Mycobacteria, thus participating in host protection (26). They also induce maturation of DC (75), thus facilitating priming of other mycobacteria-specific T-cells.

The TCR repertoire of lipid-specific T-cells is polymorphic and there is no evidence of unique TCR V genes imposing the recognition of antigens presented by CD1a, CD1b, or CD1c. There is also no evidence that type II NKT cells utilize a restricted TCR repertoire. Recently, using CD1b tetramers loaded with GMM, a rare population of T-cells was identified, which uses a nearly invariant TCR (76). These T-cells were defined as mycolyl lipid-reactive (GEM) cells. The TCR of these GMM-specific and CD1b-restricted T-cells is constituted by a TRAV1.2 gene rearranged to TRAJ19 segment. Furthermore, these T-cells showed a strong bias for usage of TRB6-2 gene and high affinity for the CD1b–GMM complex. The analysis of nucleotides in the CDR3 of the Vα chain showed that similar CDR3 amino acids were derived from different patterns of exonucleolytic trimming of the V and J regions, suggesting an antigen recognition-induced mechanism driving the expansion of these cells. This conclusion was also supported by the finding of identical nucleotide sequences in GMM-specific T-cells. In addition, the same sequences were found several times in the same donors, indicating *in vivo* expansion of GEM T-cells. A second study also showed that CD1b–GMM tetramers may identify another population of T-cells, using the TRAV17 and TRBV4-1 genes, which is present in several donors, has intermediate affinity for the CD1b–GMM complexes and shows biased but different CDR3 motifs (77). Whether this second population is expanded in donors with active or latent tuberculosis remains to be defined.

The identification of lipid-specific T-cells has made an incredible technical jump by using the tetrameric forms of soluble CD1 molecules loaded with lipid antigens. In addition to the CD1b tetramers that have been discussed above, also CD1c (78) tetramers have been generated. With the use of these tetramers it was possible to identify a population of CD1c-restricted and mycoketide-specific T-cells that use a polyclonal TCR repertoire. The identification of CD1-restricted T-cells using antigen-loaded CD1 tetramers has important implications, since detection of lipid-specific T-cells might represent a novel approach to monitor the T-cell response in patients with active or latent forms of tuberculosis.

An important set of data indicates that group 1 CD1-restricted T-cells have important functions in animal models and in humans.

Immunization of guinea pigs (*Cavia porcellus*), a species expressing multiple CD1b and CD1c gene orthologs, induces strong proliferative and cytotoxic responses restricted by CD1b and CD1c (79, 80) and immunization with total *M. tuberculosis* lipids induced a significant reduction of lung lesions (81).

Cattle (*Bos taurus*) are sensitive to natural infection with *M. bovis*, causing bovine tuberculosis that shares many clinical and pathological characteristics with human tuberculosis. Polyclonal T-cells isolated from naturally infected animals showed specific response to challenge with lipids from *M. bovis* and *M. avium paratuberculosis* (82). Uninfected animals immunized with GMM also developed GMM-specific CD1b-restricted T-cells, which persisted 4 months after the last immunization (83).

Studies performed in CD1 transgenic mice showed lipid-specific T-cells upon immunization with *M. tuberculosis* lipids or infected with *M. tuberculosis* (84).

Several studies investigated the response of human T-cells to stimulation with mycobacterial lipids *ex vivo*. Using this approach, T-cells specific for the mycobacterial antigens GroMM (22), GMM (85), SGL (26), MA (74), and MPM (30) were identified. In several cases, these T-cells were detected only in tuberculosis patients and not in healthy individuals, indicating that priming with lipid antigens occurs during infection.

A series of important immunological questions on the role of lipid-specific T-cells in tuberculosis remain to be answered. Firstly, most of the studies were performed with established T-cell clones using different cellular assays including cell proliferation, IFNγ release, and cell cytotoxicity. These assays detect protective anti-microbial capacities and indicate potential anti-microbial protective effects of these cells. These studies should be complemented with the functional analysis of T-cells freshly isolated from tuberculosis patients and possibly by *in vivo* studies using CD1 transgenic mice. Secondly, the mechanisms of lipid-specific T-cell memory generation remain unknown. The experimental data showing absence of mycobacterial lipid-specific T-cells in non-infected individuals supports that naïve T-cells are primed by a specific antigen and expand *in vivo*. Whether these cells persist for a long period of time and rapidly expand upon challenge with the antigen is not clear. These issues are of paramount importance to understand the possible use of lipid antigens as novel sub-unit anti-mycobacterial vaccines. Thirdly, the tissue-specific expression of CD1 proteins might represent a limitation to the protective activity of CD1-restricted T-cells. Macrophages are the cells harboring Mycobacteria during infection and do not express detectable levels of CD1 molecules. Therefore, these cells might be invisible to lipid-specific T-cells, thus reducing their potential protective role. Another important issue is whether CD1-restricted T-cells massively migrate to infected tissues, mostly the lung, and therefore are rare in circulating blood. The accumulation of lipid-specific T-cells in the lung of tuberculosis patients has not been properly investigated yet.

The second population of lipid-specific T-cells is represented by iNKT cells, which are innate-like T-cells. These cells are constantly activated by a variety of self- and microbial antigens and show a phenotype of pre-activate cells. They contain in their cytoplasm preformed cytokines mRNA, including IFNγ mRNA. They readily secrete these cytokines upon antigen recognition at levels that are up to 200-fold higher than those of naïve peptide-specific T-cells (86, 87). They also rapidly induce DC maturation, which in turn facilitates priming of CD8^+^ cytotoxic T-cells (88). Considering that this latter T-cell population is relevant in protection during mycobacterial infections (89), iNKT cell engagement might be also important for protection.

*In vivo* studies conducted in mice also showed that iNKT are activated during mycobacterial infections. Following BCG infection, there is an increase of iNKT cells in the lung that show signs of activation (90). iNKT cells were assumed to be important in development of lung granuloma following infection (90–92). Instead a series of studies performed using CD1d-deficient mice (lacking all types of CD1d-restricted T-cells) or Jα18-deficient animals (lacking mainly iNKT cells), did not show relevant difference in the mycobacterial colony forming units in the lung, nor in the survival of infected animals as compared to wild type mice (93–96).

Overall these studies showed that iNKT cells do not have an impact on chronic infection in mice and suggest that they are not involved in direct protective roles during tuberculosis. Thus, the potentially protective functions of iNKT cells, if any, may be present only in the early acute phases of microbial infections and be neglectable in the natural course of prolonged chronic infections such as tuberculosis.

Studies conducted in humans are observational and are limited to the numbers, phenotype, and *ex vivo* function of iNKT cells isolated from peripheral blood. None of these investigations supported an important role of iNKT cells in tuberculosis, although reduction in their number and phenotype has been observed in patients with active tuberculosis (97–99).

## Conclusion

From the overview of the studies reported here, there are increasing evidences of the relevance of the immune response elicited by mycobacterial lipids. The identification of lipid molecules essential for bacterial virulence together with the absence of functional CD1 polymorphisms is the basis for vaccine development. There is therefore increasing hope that the inclusion of lipid antigens in new vaccine formulations would lead to a successful vaccination against tuberculosis.

## Conflict of Interest Statement

The authors declare that the research was conducted in the absence of any commercial or financial relationships that could be construed as a potential conflict of interest.
